# Investigation of CRS-associated cytokines in CAR-T therapy with meta-GNN and pathway crosstalk

**DOI:** 10.1186/s12859-022-04917-2

**Published:** 2022-09-13

**Authors:** Zhenyu Wei, Qi Cheng, Nan Xu, Chengkui Zhao, Jiayu Xu, Liqing Kang, Xiaoyan Lou, Lei Yu, Weixing Feng

**Affiliations:** 1 College of Intelligent Systems Science and Engineering, Institute of Intelligent System and Bioinformatics, Harbin Engineering University, Harbin, 150001 China; 2 Shanghai Engineering Research Center of Molecular Therapeutics and New Drug Development, School of Chemistry and Molecular Engineering, Institute of Biomedical Engineering and Technology, East China Normal University, No. 3663 North Zhongshan Road, Shanghai, 200065 China; 3 Shanghai Unicar-Therapy Bio-Medicine Technology Co., Ltd, Shanghai, China

**Keywords:** CAR-T therapy, Cytokine release syndrome, Meta-learning graph neural network, Functional enrichment analysis, Pathway crosstalk

## Abstract

**Background:**

Chimeric antigen receptor T-cell (CAR-T) therapy is a new and efficient cellular immunotherapy. The therapy shows significant efficacy, but also has serious side effects, collectively known as cytokine release syndrome (CRS). At present, some CRS-related cytokines and their roles in CAR-T therapy have been confirmed by experimental studies. However, the mechanism of CRS remains to be fully understood.

**Methods:**

Based on big data for human protein interactions and meta-learning graph neural network, we employed known CRS-related cytokines to comprehensively investigate the CRS associated cytokines in CAR-T therapy through protein interactions. Subsequently, the clinical data for 119 patients who received CAR-T therapy were examined to validate our prediction results. Finally, we systematically explored the roles of the predicted cytokines in CRS occurrence by protein interaction network analysis, functional enrichment analysis, and pathway crosstalk analysis.

**Results:**

We identified some novel cytokines that would play important roles in biological process of CRS, and investigated the biological mechanism of CRS from the perspective of functional analysis.

**Conclusions:**

128 cytokines and related molecules had been found to be closely related to CRS in CAR-T therapy, where several important ones such as IL6, IFN-γ, TNF-α, ICAM-1, VCAM-1 and VEGFA were highlighted, which can be the key factors to predict CRS.

**Supplementary Information:**

The online version contains supplementary material available at 10.1186/s12859-022-04917-2.

## Background

Cellular immunotherapy is a new and effective treatment for tumor diseases. The representative cellular immunotherapy is chimeric antigen receptor T-cell (CAR-T) therapy. CAR-T therapy combines tumor associated antigen binding domain (usually a single-chain variable fragment, scFv) with the killing mechanism of T cells through gene conduction technique [1]. The produced CAR-T cells are then infused back into the patient’s body to exert their immune function and eliminate tumor cells. The therapy has shown excellent clinical effects in the treatment of refractory B-cell leukemia and other diseases [2].

However, implementation of CAR-T therapy can be associated with serious or even fatal side effects, collectively termed cytokine release syndrome (CRS). CRS is the direct result of excessive production of inflammatory cytokines caused by immune system activation beyond the physiological level. When CAR-T cells make contact with a target antigen, their intracellular structure and co-stimulatory molecules CD28/CD137 are activated. CAR-T cells proliferate and rapidly become activated within a short period of time to secrete large numbers of cytokines including interleukins, tumor necrosis factor, and interferons [3, 4]. At the same time, macrophages, monocytes, dendritic cells, natural killer (NK) cells, and other cells are activated through stimulation by the cytokines secreted from CAR-T cells. The further release of inflammatory cytokines by these activated cells can be directly or indirectly involved in the immune response. However, once the severe immune response is excessive, the body’s normal tissues and blood vessel epithelial cells are destroyed and the organs of patients can become damaged and even threaten their life [5].

Specifically, CAR-T cells can induce a systemic inflammatory response in vivo and cause CRS [6]. The activated T cells release cytokines and chemokines (including IL-2, soluble IL-2Rα, IFN-γ [IFNG], IL-6, soluble IL-6R, and GM-CSF), as do bystander immune cells like monocytes and/or macrophages (secreting IL-1RA, IL-10, IL-6, IL-8, CXCL10 [IP-10], CXCL9 [MIG], IFN-α, CCL3 [MIP-1α], CCL4 [MIP-1β], and soluble IL-6R), dendritic cells, and other cells [7, 8]. Teachey et al. [8] investigated the cytokine profiles of 51 patients with refractory B-cell leukemia, comprising 39 children and 12 adults. Among the 24 cytokines examined, the peak levels of IL-6, IL-8, IL-6R, MCP-1, and IFN-γ in patients with grade 4–5 CRS were significantly higher than those in patients with grade 0–3 CRS. Furthermore, the levels of IL-6 and IFN-γ in patients with grade 4–5 CRS were significantly higher than those in patients with grade 0–3 CRS [8, 9]. Hay et al. [10] analyzed 133 adult patients with ALL, NHL, and CLL who received CAR-T therapy. Within 36 h after infusion, the concentrations of IFN-γ, IL-6, IL-8, IL-10, IL-15, MCP-1, TNFRP55, and MIP-1R was higher in grade 4 CRS patients than in other grade CRS patients. However, because of the complexity of the human immune system, the conclusions of these studies were not sufficiently comprehensive.

Given the large number of cytokines involved in the immune response to CAR-T therapy and the occurrence of CRS, it is necessary to comprehensively predict the cytokines associated with CRS in the human body to understand the mechanism of CRS. Based on the known CRS-associated cytokines in CAR-T therapy, we used a semi-supervised meta-learning graph neural network model to comprehensively predict the CRS-associated cytokines based on big data for human protein interactions and the similarity of protein interactions. The clinical data of 119 patients who received CAR-T therapy were then used to verify the rationality of the analysis results. Finally, through protein interaction network analysis, functional enrichment analysis, and pathway crosstalk analysis, we systematically explored the roles of the predicted cytokines in the occurrence of CRS. The results show great significance for understanding the mechanism of CRS in CAR-T therapy and designing targeted blocking measures.

## Methods

### Meta-learning graph neural network

Meta-learning has made great progress in the field of image and text processing [11]. The present study used meta-learning to analyze non-Euclidean graphic data. It was reported that a graph neural network was also suitable for analysis of graphic data [12]. In this study, the meta-learning graph neural network architecture Meta-GNN was used to construct a semi-supervised classification model [13]. Through learning a small number of known CRS-associated cytokines, we aimed to achieve a comprehensive prediction of CRS-associated cytokines.

First, a human protein interaction knowledge map is constructed based on human protein interaction big data, and the knowledge map information is expressed as G = (V, E, A, X), where V is the set of nodes in the map, E is the edge set of the relationship between nodes, A is the adjacency matrix, a_*ij*_ is the weight of the connection edge e_*ij*_ of node V_*i*_ and node V_*j*_ (the weight of 0 indicates no connection), X is the feature matrix, and X_*i*_ represents the characteristics of node V_*i*_.

The Meta-GNN model includes two parts sharing the same GNN architecture: ‘meta-learner’ and ‘base-learner’. First, the meta-learner learns and optimizes the initial training parameters of the GNN model by a ‘learning to learn’ strategy, to ensure that the model has a good generalization ability. The final GNN model is then obtained through ‘base-learner’ training.

**Fig. 1:**
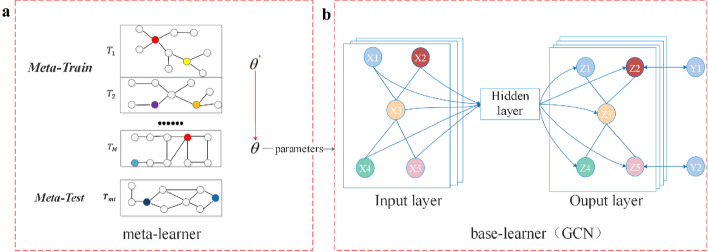
Meta-GNN is composed of two modules. **a** Meta-learner module can optimize the initial training parameters of the model. **b** Base-learner module is GCN model

The GNN model utilized in this study is a second-order GCN model. Each target node aggregates the node information of the second-order neighborhood. The main process is shown in Fig. 1. The red arrow indicates use of the Adam method for gradient descent to optimize the model parameters, ${\uptheta }\prime$ and ${\uptheta }$ represent the parameters after one and all meta-updates, respectively, and M is the number of meta-learning tasks.

The training set *D*_*train*_ is constructed based on a few nodes *(X, Y)* with labels. The meta-learning includes two steps: model training and model testing.

In the model training stage, several nodes are randomly sampled from each class of the training set *D*_*train*_ and set as *S*_*i*_. The remaining nodes in the training set are then set as query set *Q*_*i*_. The meta-learning task *T*_*i*_ = *S*_*i*_ + *Q*_*i*_ is constructed. The above steps are repeated *M* times to generate *M* meta-learning tasks.

Let *θ* be the model parameter (including weight W and deviation b of the neural network). In the implementation of the meta-learning task *T*_*i*_, the objective function is the cross-entropy function with a known label node prediction value.

1 $$\mathop L\nolimits_{{T_{i} }} (f_{\theta } ) = - \left( {\sum\limits_{{x_{is} ,y_{is} }} {y_{is} \log f_{\theta } (x_{is} ) + (1 - y_{is} )\log (1 - f_{\theta } (x_{is} ))} } \right)$$

where ${\text{x}}_{\text{is}}$ is the input vector of node ${\text{v}}_{\text{is}}$ with label ${\text{y}}_{\text{is}}$ in *S*_*i*_*.* The parameters are then optimized as:

2 $$\theta \leftarrow \theta - \alpha \frac{{\partial \sum\limits_{{T_{i} \sim p(T)}} {\mathop L\nolimits_{{\mathop T\nolimits_{i} }} (f_{{\theta_{i}^{^{\prime}} }} )} }}{\partial \theta }$$

where *p(T)* is the set of meta-learning tasks and *α* is the learning rate.

In the model testing stage, *Q*_*i*_ data are used to test the model performance for the trained model parameters to avoid overfitting problems.

The GCN model is trained after optimization. The GCN model uses graph convolution to aggregate the information of neighbor nodes. The essential purpose of the GCN model is to extract the spatial characteristics of the topological graph:

3 $$H^{(l + 1)} = \sigma \left( {\tilde{D}^{{ - \frac{1}{2}}} \tilde{A}\tilde{D}^{{ - \frac{1}{2}}} H^{\left( l \right)} W^{\left( l \right)} } \right)$$

Among them, $\widetilde{\text{A}}$= ${\text{A}}$ $\text{+}{\text{I}}_{\text{N}}$, where *A* is the adjacency matrix of the input graph and *I*_*N*_ is the identity matrix, $\widetilde{D}$= Σ_j_ ${\widetilde{A}}_{ij}$, *W*^*(l)*^ is the trainable weight, and σ(·) is the activation function. *H*^*(l)*^* ∈ R*^*N*×*D*^ is the hidden-layer embedding of nodes on an *l-*th layer, initially *H*^*(0)*^ = *X* denoting the feature matrix of nodes.

The overall forward propagation formula of the GCN model is described as:

4 $$Z = f\left( {X,A} \right) = S{\text{oftmax}}\left( {\tilde{A}{\text{ReLU}}\left( {\tilde{A}XW^{\left( 0 \right)} } \right)W^{\left( 1 \right)} } \right)$$

where *Z* denotes the output of the network, *X* is the feature matrix of nodes, *A* is the adjacency matrix, *W*^*(0)*^ and *W*^*(1)*^ are trainable weight matrices, and ReLU and Softmax are used as activation functions.

In the training process, the cross-entropy function shown in Eq. (1) is also used as the objective function. The specific training methods are the gradient descent method and the error backpropagation method.

After training, the GCN model can be used to predict the unknown class nodes in the knowledge map.

### Cytokine prediction

Human cytokine data were obtained from the NCBI website (https://www.ncbi.nlm.nih.gov/). These data include cytokines, chemokines, and soluble receptors (collectively referred to as “cytokines”), with a total of 1769 samples. The interaction data for these cytokines were then obtained from the String database (https://string-db.org/), involving 62,576 interaction data for 1615 cytokines. We also provide a supplementary document (Additional files 1, 2) with full implementation details. Based on this, a topology map is constructed to form an adjacency matrix representing the interactions between cytokine nodes. Here, the characteristic matrix of the cytokine nodes is set as the identity matrix.

Through literature mining, we obtained 20 positive data for cytokines confirmed to be associated with CRS, namely CXCL8/IL-8, IL-17A, CXCL10/IP-10, CXCL9/MIG, CCL4/MIP-1B, CCL3/MIP-1A, CCL2/MCP-1, IL-1A, IL-1B, TNF-α, IL-2, CSF2/GM-CSF, IL-2RA, IL-5, IL-15, TNFRSF1A, IL-4, IL-13, IL-23A, and ANGPT2**/**ANG2. In addition to the positive data, we selected 100 cytokines as negative data whose nodes in the topology map are the farthest from the positive ones. Since more cytokines are not associated with CRS compared to those associated with CRS, more negative data were chosen to reduce false-positives.

The adjacency matrix, characteristic matrix (identity matrix), and positive and negative label data were put into Meta-GNN for model training. After training the model, we can obtain the probabilities for all nodes associated with CRS. Keeping the positive label data unchanged, the negative label data are conducted to the last 100 data with the minimum prediction probability, and the model training is conducted again until the prediction results converge.

**Fig. 2:**
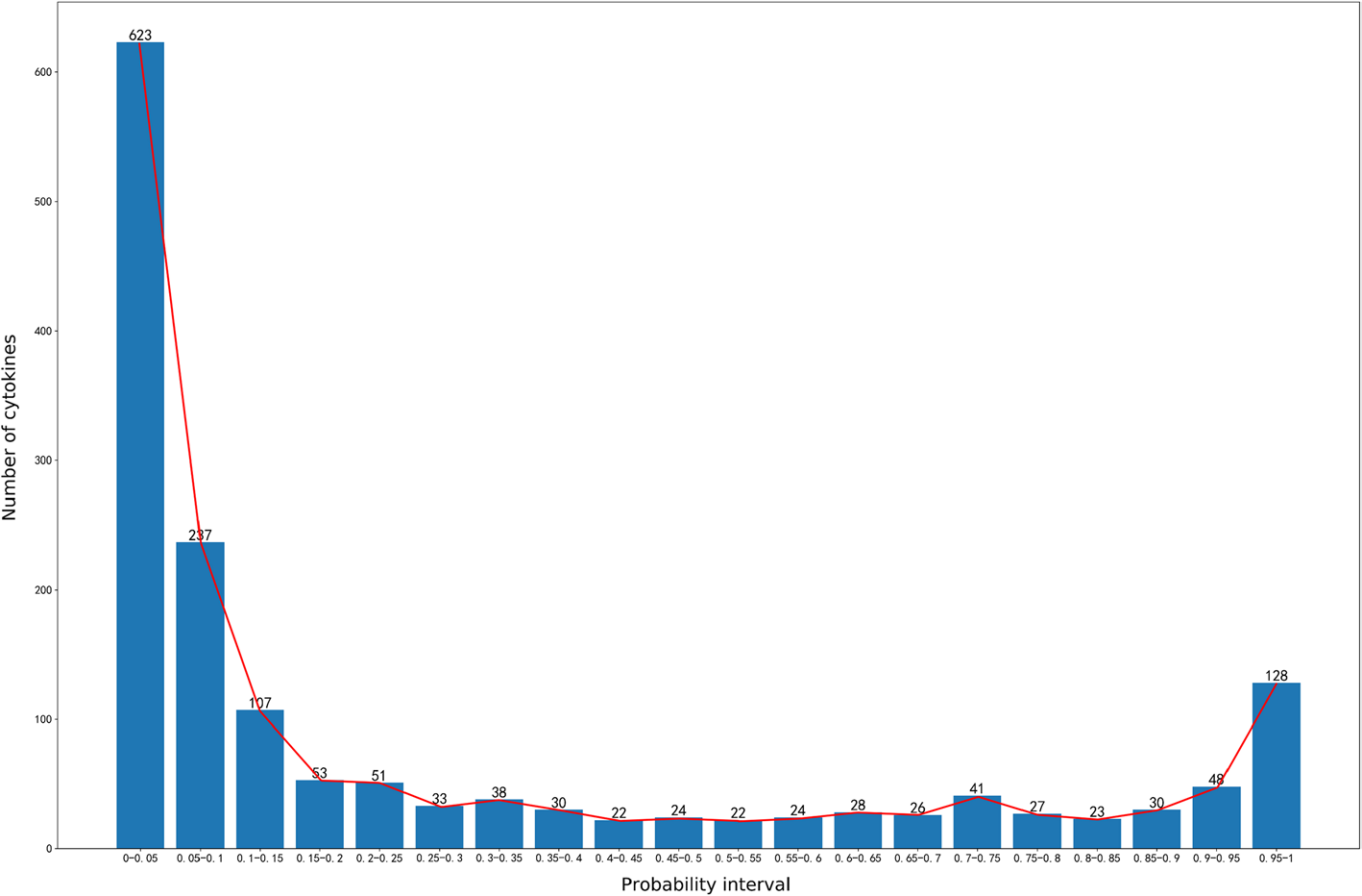
Probability histogram of the predicted results, the horizontal axis represents the value range of the prediction probability, and the vertical axis represents the number of cytokines in a certain time interval. We selected 128 cytokines with a probability greater than 0.95 at the far right as the result

To avoid any influence of the initial data selection on the prediction results, 100 negative label data are randomly selected and the above prediction process is repeated. This process is repeated 1000 times. Finally, the prediction results are arranged from large to small according to the prediction probabilities associated with CRS, and the median value of the 1000 prediction results is taken as the final prediction result for each cytokine. The prediction results are shown in Fig. 2. A total of 128 cytokines and related molecules with probability values above 0.95 were selected as the cytokines associated with CRS.

### XGBoost validation

The clinical data for 119 patients with B-ALL who received CAR-T therapy were collected, and the details of data were described in Table 2. The data were grouped according to CRS levels.

XGBoost [14] algorithm was always used for classification analysis. We evaluated the contribution of each type of data to CRS with it.

### Functional enrichment analysis

We carried out KEGG pathway enrichment analysis and GO enrichment analysis on the predicted cytokines, collectively referred to as functional enrichment analysis [15, 16]. Through functional enrichment analysis, we not only found out the pathways such as MAPK, NF-KB, JAK-STAT3 and mTOR which were found in previous authoritative studies [17], but also explained the combination of other pathways, such as VEGF pathway and NOD-like receptor pathway. Through the analysis of these pathways, we tried to explore how CRS happened, which makes people more clearly understand what the mechanism of CRS happened. Cytokines with high frequency in these pathways also indicate the importance of influencing CRS, such as IFN-γ, IL1B, IL6 and TNF-α mentioned above. These factors echo with vascular inflammatory factors ICAM-1, VCAM-1 and VEGFA in VEGF pathway, and jointly promote the progress of CRS.

### Pathway crosstalk analysis

Based on the enrichment analysis of CRS-related cytokines, the crosstalk of CRS-related cytokines was analyzed. The analysis is based on the assumption that two pathways sharing a certain proportion of cytokines are considered to have crosstalk [9, 18].

To describe the degree of crosstalk between a pair of pathways, two measurements can be calculated, namely the Jaccard coefficient (JC)$= \left| {\frac{A \cap B}{{A \cup B}}} \right|$ and the overlap coefficient (OC)$= \frac{{\left| {A \cap B} \right|}}{{\min \left( {\left| A \right|, \left| B \right|} \right)}}$, where A and B are the lists of cytokines contained in the two pathways.

The specific steps are as follows:

1. For selection of a pair of enrichment pathways, the FDR value of each enrichment pathway should be less than 0.05 and the pathways should contain more than 5 enriched cytokines, because too few cytokines may lack sufficient biological information.
2. The number of associated cytokines shared between the two pathways is calculated. Any pathway pair with less than 4 shared cytokines is removed.
3. For all eligible pathway pairs, the mean value of the sum of the JC coefficient and the OC coefficient is calculated.
4. To reduce false-positives, pathways with a mean value above 0.3 are selected for analysis.
5. Visual screening of the pathway pairs is performed, with intuitive display of their crosstalk [19, 20].

## Results

### Prediction results

The results predicted by the Meta-GNN model are expressed in the form of probabilities. High probability means more similarity to the positive label data, indicating a closer relationship to CRS. The predicted results were shown in Table 1. Altogether, 128 proteins were found to be closely associated with CRS in CAR-T therapy.

**Table 1:** Prediction results

Name	Scores	Name	Scores	Name	Scores
IL10	0.999	ITGAX	0.989	CCL17	0.975
IL4	0.999	VCAM1	0.989	TLR5	0.975
IL17A	0.999	IL1RN	0.989	STAT3	0.975
CSF2	0.999	IL6R	0.988	MYD88	0.975
IL13	0.999	CCR6	0.988	PTPRC	0.974
IFNG	0.999	TLR2	0.988	TLR6	0.974
IL5	0.999	FOXP3	0.987	IL12RB1	0.973
CSF3	0.998	IL22	0.987	CX3CR1	0.973
IL15	0.998	IL17F	0.987	IL17RA	0.973
CXCL10	0.998	CXCL5	0.986	PRF1	0.972
CXCL8	0.998	CTLA4	0.985	CCL4L1	0.972
CCL2	0.998	SELL	0.985	CRP	0.972
IL2	0.997	IL33	0.985	CX3CL1	0.971
IL7	0.997	CCL11	0.985	TNFRSF1A	0.97
CCL3	0.997	TLR9	0.985	IFNB1	0.969
IL18	0.996	CXCL12	0.985	MMP9	0.969
CXCL9	0.996	OSM	0.985	CCL27	0.968
TNF	0.996	CD28	0.984	CCL22	0.968
IL1B	0.996	CXCR4	0.984	SELP	0.967
CCL4	0.996	LTA	0.983	IL3RA	0.967
CCL5	0.995	TSLP	0.983	CD274	0.966
IL1A	0.995	IFNA1	0.983	STAT5A	0.965
CXCL1	0.995	STAT1	0.983	TLR10	0.965
CD40	0.994	TLR7	0.983	IL2RB	0.964
ICAM1	0.994	CXCL13	0.982	JAK1	0.964
CCL20	0.994	IL23A	0.981	CCL8	0.963
CXCL2	0.994	CCR1	0.981	IL12B	0.962
CCR2	0.993	TLR8	0.981	CSF1	0.962
IL2RA	0.993	IL11	0.98	CCL21	0.96
IL9	0.993	TLR4	0.98	IL1R2	0.96
IL1R1	0.993	IL21	0.979	IL7R	0.958
CD86	0.992	IL23R	0.979	IDO1	0.958
IL3	0.992	TLR1	0.979	IL2RG	0.958
CD80	0.992	CXCL11	0.979	GZMB	0.958
CCR7	0.991	TBX21	0.978	CD1C	0.957
CD40LG	0.991	TNFRSF1B	0.978	TNFRSF4	0.956
IL6	0.991	TLR3	0.978	CD83	0.956
IL10RA	0.991	IL16	0.978	STAT6	0.955
CCR5	0.99	CXCR5	0.977	TNFSF13B	0.955
IL4R	0.99	CCL19	0.977	CCR9	0.953
ITGAM	0.99	CD19	0.976	CXCR1	0.953
CXCR3	0.99	CXCR2	0.976	JAK2	0.952
SELE	0.989	CCL7	0.976		

### Clinical data validation

The clinical data collected from 119 patients includes four categories: cytokine expression data, coagulation test data, biochemical test data, and blood routine test data (Table 2). Before contribution analysis with XGBoost, three cytokines closely related to CRS(IL10, IL6 and IFN-γ) were taken out, which were not regarded as positive data. According to the analysis results, these three cytokines are all top ranked in the list, indicating the reliability of the prediction results.

**Table 2:** Clinical data of patients with acute lymphoblastic leukemia

Cell factor	IL-2、IL-4、IL-6、IL-10、TNF-α、IFN-γ、IL-17A
Coagulation	Plasma prothrombin time, activated partial thromboplastin time, fibrinogen
Biochemistry	Gamma-glutamyl transpeptidase, lactate dehydrogenase, creatinine, C-reactive protein, ferritin, sodium, potassium, chlorine, calcium, uric acid, glucose, triglyceride, albumin, alanine aminotransferase, aspartate aminotransferase, alkaline phosphatase
Routine blood test	White blood cells, hemoglobin, red blood cells, neutrophil percentage, hemoglobin count, neutrophil count, lymphocyte percentage, blood routine items, red blood cell count, lymphocyte count, platelet, monocyte percentage, monocyte count

In the process, we calculated mean values of all data from day 0 of CAR-T therapy to the last day of records which is about 7 days to analyze the relationship with the CRS levels of the patients.

**Fig. 3:**
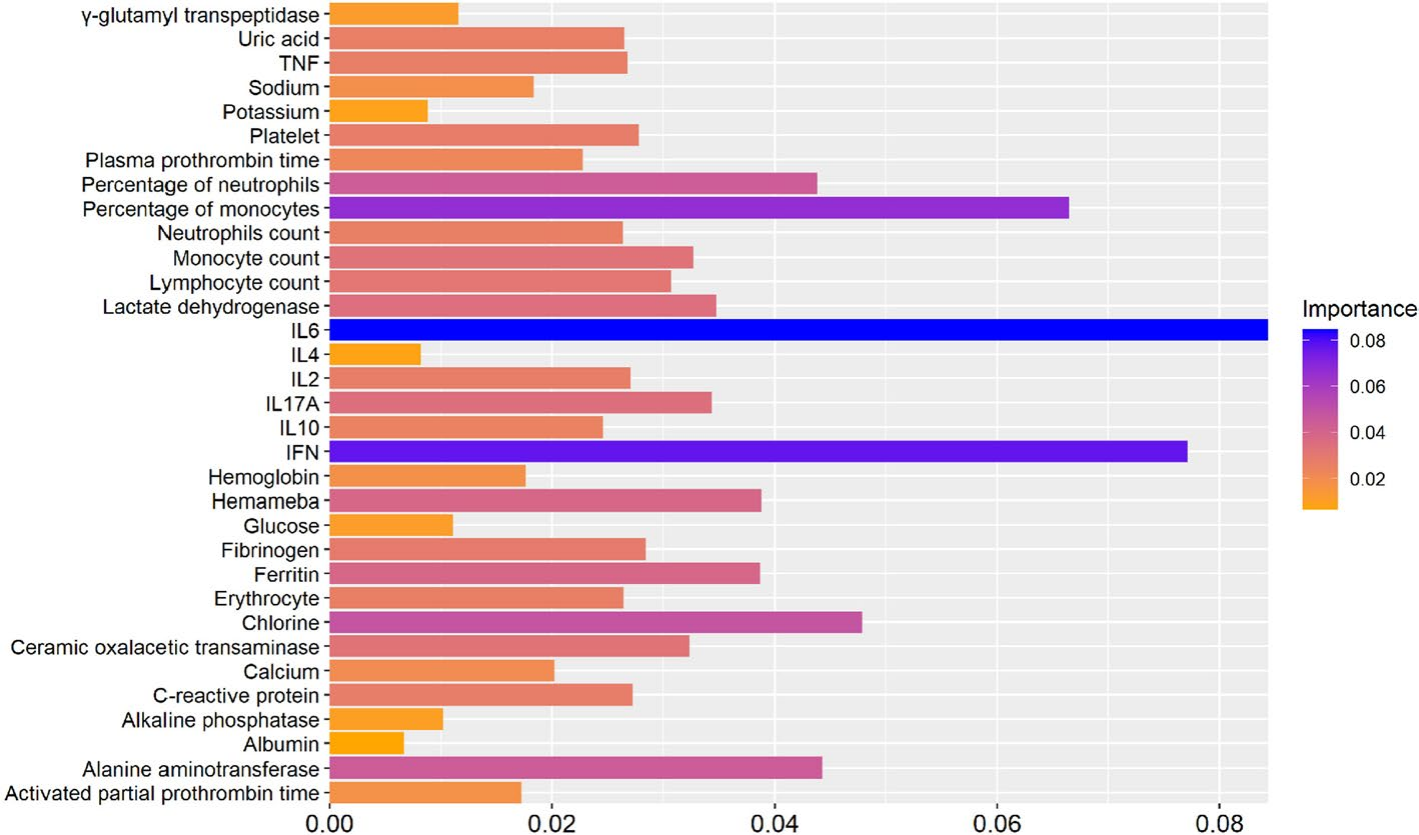
The XGBoost algorithm ranks the features importance of the features data used to predict CRS

The XGBoost algorithm was used for classification analysis, and the contribution of each type of data to the highest level of CRS prediction was analyzed. A fivefold cross-validation was used in the research, and the results are represented by the importance of features, as shown in Fig. 3. A high probability indicates the corresponding data contribute greatly to CRS prediction (Additional file 1).

As shown in Fig. 3, IL-2, IL-6, IL-10, TNF-α, IFN-γ, and IL-17A all contribute to prediction of the highest CRS level, except for IL-4. Among them, IL-6 contributed the most to the results, IL10 and IFN-γ had a significant contribution. This indicates that three cytokines are positively correlated with CRS. Although they are not regarded as positive label data, they rank higher in the prediction results of the neural graph networks, thereby proving the rationality of the prediction results (Additional file 2).

We also included some enzymes in the results. The enzymes are associated with organ damage. For example, high levels of LDH are often found in patients with grade 3–4 neurotoxic lymphoma and are negatively correlated with progression-free survival [21]. These findings are consistent with the symptoms of CRS.

### Function analysis

First, we performed a network analysis on 128 selected cytokines and related molecules. Subsequently, a pathway analysis and GO enrichment analysis were performed. Fisher’s exact test was used to show the overlapping significance between pathways and input genes. In the analysis results, FDR values less than 0.05 were considered to indicate significant enrichment. The results showed the main pathways and biological functions of the cytokines. Finally, the cytokine pathways were extracted for crosstalk analysis and further explanation of how CRS occurs from an intuitive perspective.

### Protein interaction network analysis

**Fig. 4:**
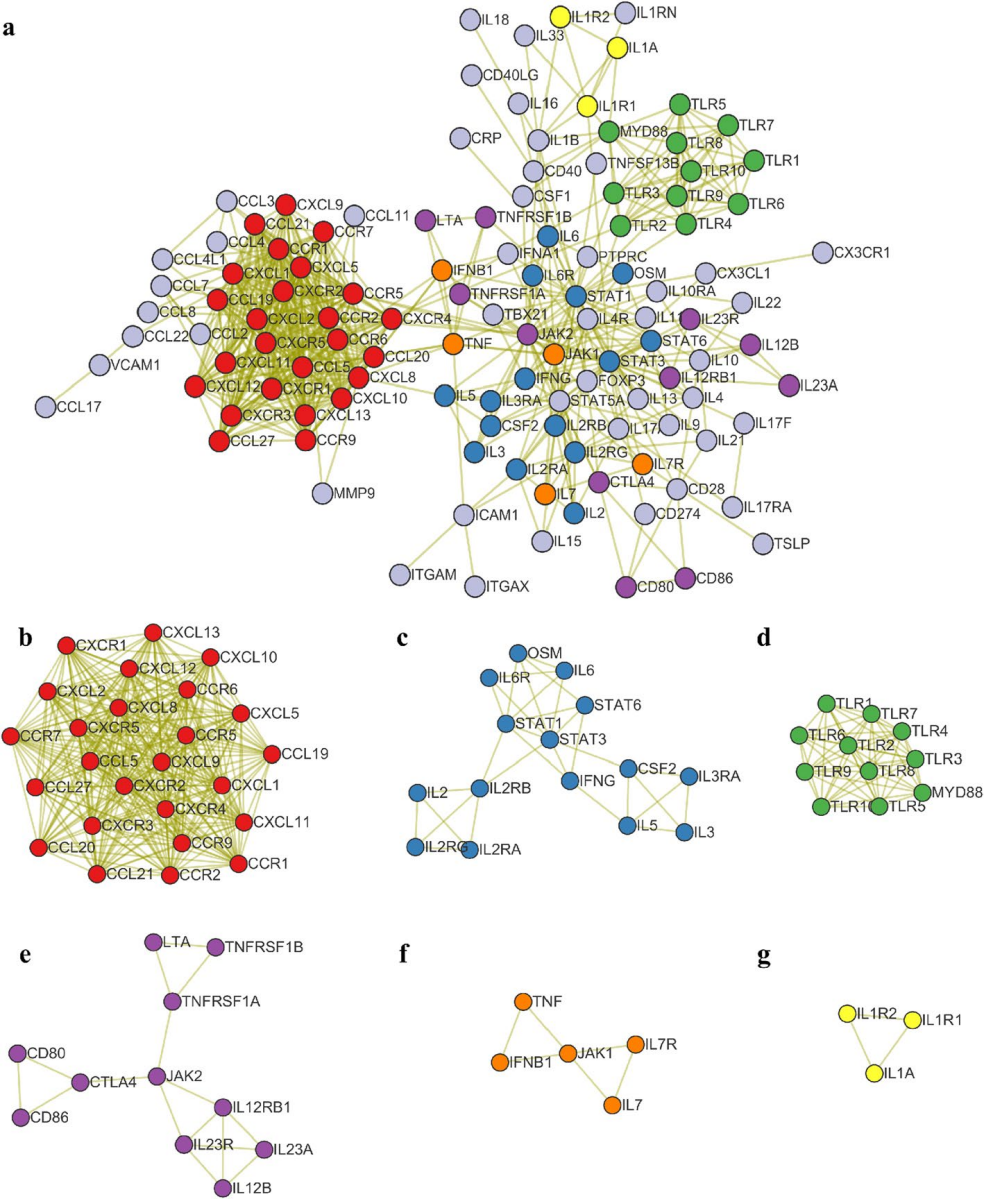
Cytokine interaction network. **a** Network interaction of cytokines. **b** Chemokine network. **c** Interleukin and its receptors and signal transduction factors. **d** Toll-like receptors. **e** JAK1 connected module. **f** JAK2 connected module. **g** IL-1 and its receptor

A network interaction diagram of the 128 cytokines and related molecules was drawn using Cytoscape (Fig. 4). As shown in Fig. 4a, the interaction diagram can be roughly divided into left and right modules. The left module is mainly composed of chemokines, and the right module is primarily composed of interleukins and soluble receptors.

The cytokines contained in each part are closely related and closely linked. Figure 4b shows the part involving chemokines. Chemokines are types of cytokines that promote various immune cell functions, including leukocyte recruitment and transportation. Transport disorder in the inflammatory process is related to an excessive inflammatory response. Figure 4c mainly includes IL-2Rα/IL-2RA, IFN-γ/IFNG, IL-6, IL-6R, and GM-CSF/CSF2 [8]. This module corresponds to many cytokines involved in the CRS response. Soluble IL-6 binds to IL-6R to form IL-6 and IL-6R complexes that bind to gp130, and subsequently initiate signal transduction through the intracellular domain. Signal transduction is mediated by the JAK-STAT3, PI3K-AKT, and MAPK pathways [17]. Figure 4d shows Toll-like receptors (TLRs). The interactions between TLRs and their ligands induce cascade activation through the TIR domain and various mediators, such as myeloid differentiation protein 88 (MyD88), TIR domain-containing protein (TIRAP), TIR receptor-induced interferon (TRIF), TRIF-related molecule (TRAM), and some tyrosine kinases. Because of these signaling cascades, transcription factors like NF-κB can induce inflammatory cytokine production [22]. Figure 4e, f show modules connected by JAK1 and JAK2. Among them, IL-23, which is closely related to CRS, comprises IL-23A and IL-12B shared with IL-12. IL-23 is related to Crohn’s disease, rheumatoid arthritis, psoriasis, and other immune-mediated inflammatory diseases [23]. TNF is an effective multi-functional proinflammatory cytokine belonging to the superfamily comprising TNF and its receptors such as TNFRSF1A and TNFRSF1B. In addition to inducing fever, enhancing systemic inflammation, and activating antimicrobial responses (such as IL-6 production), TNF can also induce apoptosis and regulate immunity. TNF and other cytokines in the TNF and TNF receptor superfamily are effective inducers of NF-κB, which leads to the expression of a variety of pro-inflammatory genes [17]. Figure 4g shows IL-1 and its receptors. RNAseq data for myeloid cell types collected at the onset of CRS revealed that IL-1R1 was up-regulated in tumor-associated myeloid cells, while only IL-1R2 was detected in spleen myeloid cells [9]. Blockade of IL-1 can reduce CRS and neurotoxicity [24].

These six modules are not isolated from one another. Instead, they are closely related to a large module through certain cytokines.

### Functional enrichment analysis

**Fig. 5:**
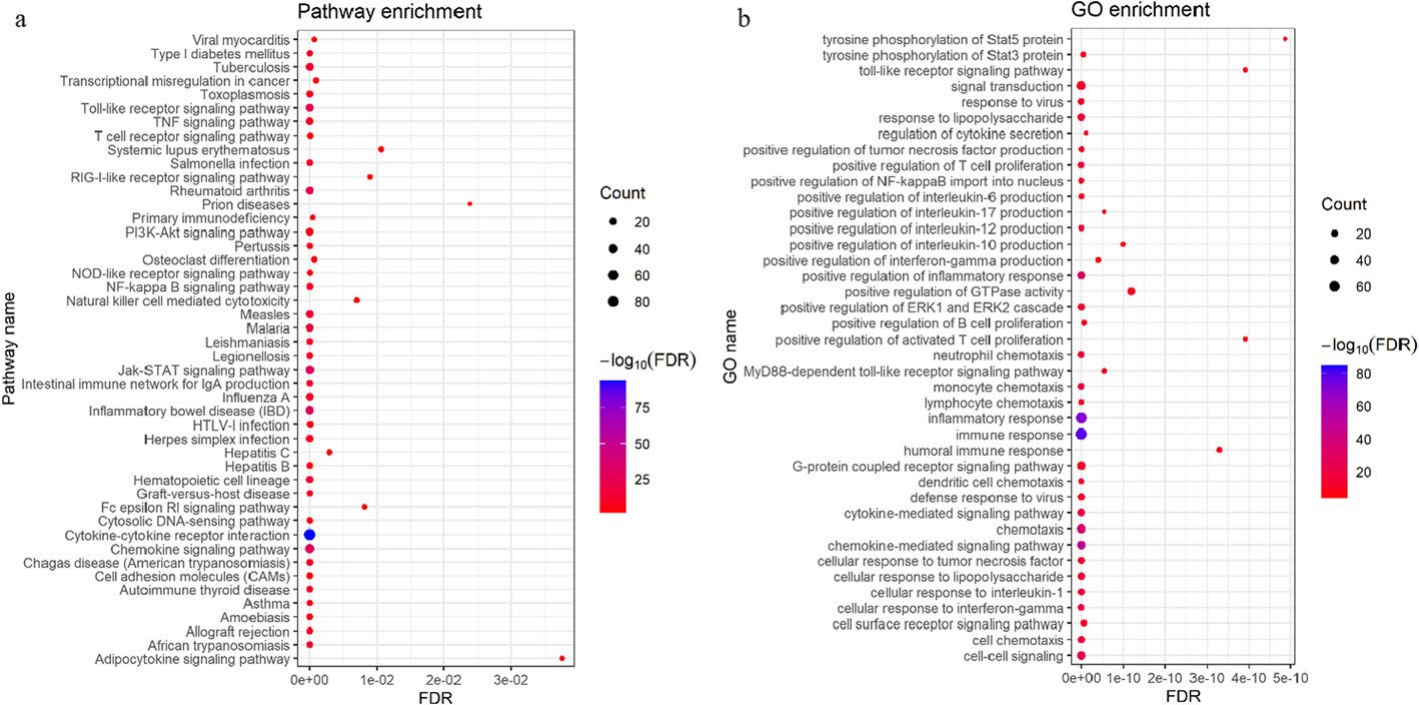
Functional enrichment analysis of bubble diagram. **a** Represent the pathway enrichment analysis, the horizontal axis represents the FDR value after logarithm base 10, and the vertical axis represents the pathway name. **b** Denotes GO enrichment analysis, the horizontal axis also denotes FDR value after logarithm base 10, and the vertical axis denotes the biological process of enrichment

The pathways and major biological processes involving the cytokines were enriched. In the pathway analysis, a total of 49 pathways were enriched. Forty-seven important pathways with FDR values below 0.05 were selected, as shown in Fig. 5a. Among the biological processes, we chose the first 40, as shown in Fig. 5b. Colors corresponding to low FDR values tend toward blue, while colors corresponding to high FDR values tend toward red. The circle sizes indicate the numbers of cytokines contained. The selected pathways and biological processes provide important information for understanding the mechanism of CRS.

As shown in Fig. 5a, most of the selected pathways are related to the inflammatory response and related diseases, and several of them are particularly prominent. For example, “cytokine receptor interaction pathway” is the most important, and reflects an essential pathway in life activities. This pathway is closely related to cancers, autoimmune diseases, metabolic disorders, and other diseases [25]. The JAK-STAT signaling pathway, which is connected with the first pathway, is an essential cytokine signal transduction pathway. It is activated by various cytokines, growth factors, and receptors and participates in cell proliferation, differentiation, apoptosis, angiogenesis, and immune regulation [18, 20]. Other inflammatory disease-related pathways were also selected.

In Fig. 5b, many terms related to the human inflammatory response are selected, including positive regulation of chemokine response, immune response, and cell response to lipopolysaccharide. These findings are consistent with the fact that CRS is an inflammatory response.

### Pathway crosstalk analysis

To further highlight the details of the pathway enrichment and understand the interactions between pathways, we conducted pathway crosstalk analysis among the 47 pathways with significant enrichment. Among these, 45 pathways met the crosstalk analysis standard. Specifically, each pathway shared at least four genes with one or more other pathways, and had an FDR value less than 0.05. To reduce the false-positive rate, we selected the pathway pairs with mean JC and OC values above 0.3. A total of 306 pathway pairs were reserved from the 45 pathways.

**Fig. 6:**
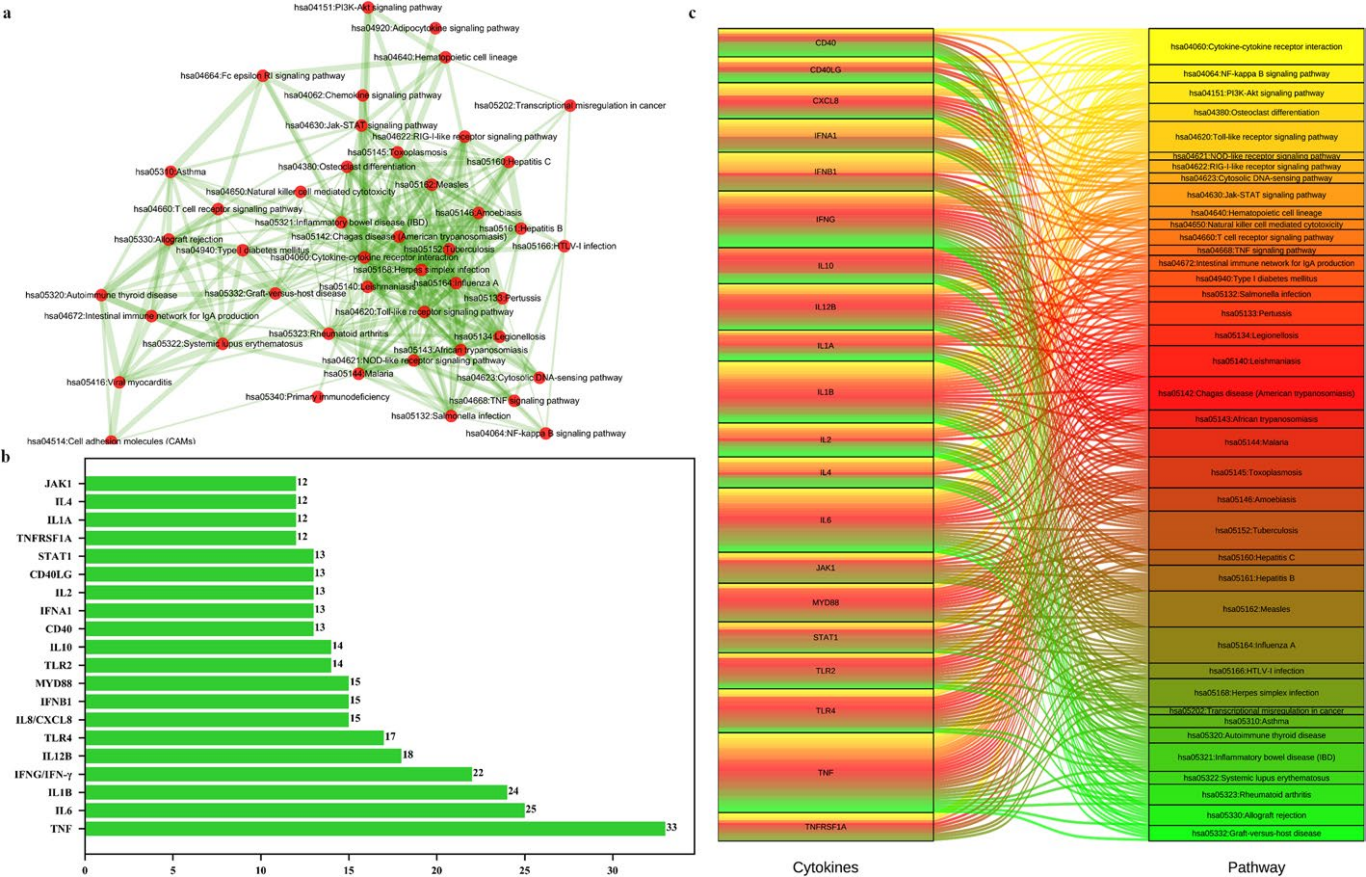
Pathway crosstalk studies of pathways and cytokines. **a** Interactions of 45 channels in channel crosstalk. **b** The top 20 cytokines with the highest frequency appeared in this pathway. **c** Sankey diagram, which shows the pathways of the top 20 cytokines in frequency

Based on their crosstalk, the pathways can be divided into left and right modules. Each module contains more interactions than other modules, indicating that they may participate in the same or similar biological processes. In Fig. 6a, the width and thickness of the figure lines are related to the degree of interaction between the pathways. A high level of interaction corresponds to a thick line, while a low level of interaction corresponds to a thin line [26–28]. The right module is mainly related to inflammatory response and disease pathways, such as PI3K-AKT signal transduction, Toll-like receptors signal transduction, NF-κB signal transduction, JAK-STAT signal pathway, inflammatory bowel disease, and hepatitis C. The left module is mainly related to immune response pathways, such as systemic lupus erythematosus, autoimmune thyroid disease, and intestinal immune network for IgA production. These findings are consistent with the side effects of CAR-T therapy. The top 20 cytokines with the highest frequencies and their involved pathways are shown in Fig. 6b, c. The high frequencies of the cytokines suggest their importance.

## Discussion

In the past few years, through the acquisition of clinical symptoms and monitoring data for patients, researchers have understood the pathogenesis of CRS produced by CAR-T therapy. With the development of high-throughput technology, certain cytokines have been gradually identified as being related to the disease. However, the biological process for comprehensive understanding of CRS pathogenesis at the molecular level has not been established. Some related cytokines have not been found. Therefore, it is necessary to explore CRS pathogenesis and related cytokines at the system biology level [29, 30].

The system analysis method described in our study has significant advantages. First, we collected a comprehensive set of human cytokines, providing a valuable source for further analysis. In addition, we used Meta-GNN as a neural graph network. This method was suitable for small sample size prediction in the study, because it can predict some cytokines that may be closely related to CRS based on known cytokines closely related to CRS as positive markers. Subsequently, we obtained data for 119 patients with B-lymphoblastic leukemia who received CAR-T therapy to allow feature screening. We applied the XGBoost algorithm to rank the importance of the measured features in our experimental data and verified the accuracy of the prediction results. Finally, we selected the positive labels and predicted the cytokines that may be closely related to CRS for subsequent analysis. We have provided a comprehensive and systematic framework to describe the biological processes and functional features of CRS produced during CAR-T therapy.

The cytokine analysis mainly showed modules that are closely related to the cytokines, such as chemokines, Toll-like receptors, and interleukins. These cytokines play an important role in the development of CRS.

The GO analysis mainly enriched the biological processes related to CRS, among which the inflammatory response was the most prominent. Examples included cytokines, chemokine-mediated responses, and cellular responses to LPS. Members of the interleukin family and chemokine family can increase the proliferation and activation of CAR-T cells, providing positive feedback for the inflammatory response. Lipopolysaccharide is considered a crucial inflammatory marker in CRS induced by CAR-T therapy [29, 30]. Besides, there is positive regulation of T cell proliferation. CAR-T therapy maintains the tumor-killing ability through T cell proliferation. A large increase in T cells is related to a high risk of CRS, consistent with previous findings.

The pathway analysis showed that the JAK-STAT signaling pathway, chemokine signaling pathway, TNF signaling pathway, Toll-like receptors pathway, and NF-κB signaling pathway were enriched. These pathways related to inflammation were enriched, and their FDR values were low. The findings suggest that many cytokines are involved in CRS, consistent with its situation in pathological development. Some disease-related pathways may contribute to the early stage of CRS, such as inflammatory bowel disease, hepatitis B, hepatitis C, and rheumatoid arthritis. The pathway interaction module is mainly divided into two modules. One module is primarily related to inflammatory response and disease pathways, and the other module is mostly related to immune response pathways and diseases caused by these pathways. Activation of a series of inflammatory and immune signaling pathways can regulate various physiological processes in the human body and cause damage to patients.

When CAR-T cells enter the human body, they directly cause the tumor cells to burn out, so that ATP, the energy molecule in the cells, is released in large quantities, and ATP can strongly activate macrophages [31]. Macrophages promote assembly of signaling complexes by inducing activation of TCR adjacent to tyrosine kinases, thereby activating downstream signaling pathways such as MAPK, PKC, and calcium ions. All of these promote the activity of the transcription factor NF-κB and regulate the expression of effector protein molecules, thus enhancing the lethality of T cells to the tumors and leading to the release of more cytokines [32]. The released cytokines activate the JAK-STAT pathway, which acts as an inflammatory signal pathway for stress, and has a rapid response. This pathway involves the IL-6ST (gp130) receptor family. IL-6 binding causes dimerization of the receptor and activates its binding of JAK protein. The activated JAK protein phosphorylates both the receptor and itself. These phosphorylation sites are the binding sites of STAT proteins and adaptor proteins with an SH2 structure. Adaptor proteins connect the receptors with MAPK, PI3K-AKT, and other pathways [33–35].

Subsequently, Toll-like receptors and NOD-like receptor (NLRs) play important roles in inflammation as extracellular and intracellular pattern recognition receptors, respectively. The TLR signal transduction pathway is activated by the Toll/IL-1R (TIR) domain, which activates the transcription factor NF-κB and MAPK pathway through MyD88. When CAR-T cells attack tumor cells, they cause the tumor cells to die and release antigens. When Toll-like receptors recognize pathogen-related molecular patterns (PAMPs), they activate NF-κB to initiate inflammatory reactions. Moreover, when inflammation occurs, LPS released by the inflammatory response induces a series of gene expressions through TLR4. The expression of antiviral cytokines such as CD80, CD86, and IFN-β is influenced by the MyD88-independent pathway, which participates in inflammation and the immune response. After the NOD1 and NOD2 proteins in the NOD-like receptor pathway bind to their ligands, they interact with RIP2 to phosphorylate IKB, thus activating the transcription factor NF-κB to mediate the expression of inflammatory mediators [36]. Activation of the NF-κB pathway regulates the expression of a series of genes, including ICAM-1, VCAM-1, SELE, TNF-α, IL-1β, IL-2, IL-6, MCP-1, IL-8, IL-12, and IFN-β, as well as some receptor molecules such as IL-2R and T cell receptor α and β chains [37–39]. TNF-α, IL-1, and other inflammatory mediators activate different MAPK pathways and mediate the inflammatory response [40, 41].

The TLR/NF-κB pathway is the critical link for the induction of inflammation. When LPS stimulates human umbilical vein endothelial cells, TLR4 expression is up-regulated, leading to increased TLR4 activity. The NF-κB component RELA plays an important role in the regulation. NF-κB1 also had a vital role in the TLR/NF-κB pathway. LPS activates NF-κB1 through TLR4 and subsequently produces a series of inflammatory mediators, leading to an inflammatory reaction that damages tissues and cells [37–39]. These observations demonstrate that there is a close relationship between CRS and this pathway.

From the perspective of vascular endothelial cells, ICAM-1, VCAM-1, and SELE bind to ligands on the surface of leukocytes to mediate leukocyte adhesion, and further induce leukocyte aggregation and infiltration. This causes local inflammation and microvascular endothelial cell injury, and subsequently promotes expression of VWF. After vascular injury, NO free radicals are released. Abnormal NO production causes vasodilation and hypotension, as common clinical features of CRS [9, 19, 20, 42]. a previous study further found that during severe CRS, endothelial cell activation leads to increased levels of biomarkers including VWF and Ang2, consistent with the manifestations of vascular instability, capillary leakage, and consumptive coagulation disorder in severe CRS [18]. Activated endothelial cells are a critical source of IL-6 in CRS. Biomarkers for activated vascular endothelial cells can help to determine which patients are at the greatest risk for CRS and neurotoxicity. In general, expression of ICAM-1 and VCAM-1 in normal blood vessels cannot be detected by standard immunohistochemical methods. However, under conditions of vascular injury or inflammatory factor stimulation, ICAM-1 and VCAM-1 are not only expressed on VECs in a time-dependent manner, but also mediate monocyte adhesion to endothelial cells and promote the development of inflammation [43]. It was reported that endothelial cells in the inflammatory reaction stage secrete vascular endothelial growth factor (VEGFA) to mediate the activation of NF-κB, thereby promoting the expression of ICAM-1 and VCAM-1. Some researchers further confirmed a regulatory effect of NF-κB on the expression of ICAM-1 and VCAM-1 and found that activation and regression of NF-κB preceded the activation and regression of ICAM-1 and VCAM-1. These observations suggest that ICAM-1, VCAM-1 and VEGFA are key factors for predicting CRS.

Regarding cell apoptosis, apoptosis of vascular endothelial cells is inhibited during the occurrence of CRS. Specifically, activation of the PI3K-AKT signal transduction pathway inhibits cell apoptosis, which has an important role in the pathogenesis and apoptosis mechanism underlying inflammation, tumor development, metabolism, and other diseases. When the PI3K-AKT signal transduction pathway becomes activated, activated Akt increases the content of NF-κB and activates the NF-κB pathway. The PI3K-AKT signal transduction pathway upregulates the transcription levels of TNF-α and other genes through a series of reactions. TNF-α promotes the production and release of other cytokines and finally leads to the formation of a cytokine network that expands the inflammatory chain reaction. The apoptosis induced by TNF-α, IFN-γ, and other cytokines depends on the PI3K-AKT pathway. Apoptosis plays an important role in the regression of CRS. The PI3K-AKT signal transduction pathway inhibits apoptosis of T cells and vascular endothelial cells and promotes continuation of an inflammatory state, providing powerful insights into the persistence of CRS [44].

The cascade reaction in CRS is one of the main reasons for the occurrence of CRS. We analyzed some of the major inflammatory pathways involved in CRS and pointed out certain cytokines and proteins that closely related to CRS. We also discussed their roles in CRS occurrence, providing important insights and assistance for studies on blocking and weakening CRS in the future.

## Conclusions

The development of CRS during CAR-T therapy is complex and related to many factors. In the present study, we applied a meta-graph neural network framework, machine learning algorithm, and system biology analysis to determine the cause of CRS and identify cytokines with a probability exceeding 0.95 in the predicted results. Through PPI analysis, functional enrichment analysis, and pathway crosstalk analysis, we identified the biological processes and pathway modules related to CRS to explain the cause of CRS. Our prediction results provide meaningful inferences for CRS and have the value of identifying potentially related cytokines. The results suggest great promise for analysis of the CRS mechanism at the system biology level. With the development of high-throughput technology and increase in medical experimentation, the actual useful cytokines by experimental results will inevitably appear in the results of our prediction and analysis.

## Supplementary Information

**Additional file 1**. Cytokines used in the study.

**Additional file 2**. Interactions between cytokines used in the study.

## Data Availability

The cytokines used in the study come from NCBI(https://www.ncbi.nlm.nih.gov/); The interactions used in the study come from the STRING database(https://cn.string-db.org/).
